# Laryngoscopy

**Published:** 1873-11

**Authors:** Beverly Robinson


Laryngoscopy.—*Beverly Robinson, M.D.*—With regard to that very troublesome affection, ozœna, Dr. Fauvel often accomplishes a cure by repeated injections of nitrate of silver into the nasal passage, beginning with dilute solutions, (one part of the salt to thirty of water), and reaching, finally, a concentrated solution made with equal parts of the salt and water.

He makes use, not of the continuous nasal douche, but of a syringe, terminating in an extremity or nozzle, perforated like that of a water-pot for watering flowers. Besides this, the tube, which screws on and off the syringe, is of such a shape that it may be introduced far back into the mouth, and up beyond the uvula and posterior limit of the soft palate, and there used to wash out the posterior nares and naso-pharyngeal cavity either with water or any medicated solution we may prefer.

Confidence is still attached to the local employment, as a gargle, of bromide of potass (10–20 grs. to oz.j) for producing anæsthesia of the throat, and thus in some cases, where there are great irritability of the fauces and troublesome reflex action, rendering less difficult what is otherwise almost impossible, the proper introduction of the laryngeal mirror. In a few cases, and these were evidently of a specially nervous type, we have seen the bromide produce the tolerance wished for.

In many others, we are equally sure that it failed completely to accomplish the desired effect. Sucking ice for ten or fifteen minutes prior to a laryngoscopic examination, or gargling the throat during a less period with ice-cold water, appears to us a better and more efficacious plan to attain the result sought after.

Iodide of potass must not be given in cases of tertiary syphilis, when the œdema is considerable, for fear lest it (œdema) may be increased rapidly, and the operation of tracheotomy be made an absolute necessity in order to save a life which would otherwise be inevitably lost.

The efficacy of the dilute mineral acids, especially nitric acid, taken internally, in cases of papillary growths in the larynx, is believed in. In fact, Dr. Fauvel finds a favorable analogy for this method in that acetic acid, nitric acid, etc., are successfully employed in *external* applications on warts. To nervous, hysterical patients, bromide of potass is largely given internally, and in these cases, if there be incomplete or complete aphonia, local applications of electricity, by means of Mackenzie's electrode, are made with great success to the cords themselves.

